# Supplementary material for: Pharmacokinetics and Dose Proportionality Study of a Novel Antiparkinsonian Agent, a 1H-1,2,4-Triazol-3-ylthio-conjugate of Prottremine
Source: Molecules. 2024 Sep 22;29(18):4498. doi: 10.3390/molecules29184498 (PMC11434290; doi:10.3390/molecules29184498)
Supplement: Supplementary file 1 [file molecules-29-04498-s001.zip › molecules-3195375-supplementary.pdf]

Supplementary materials

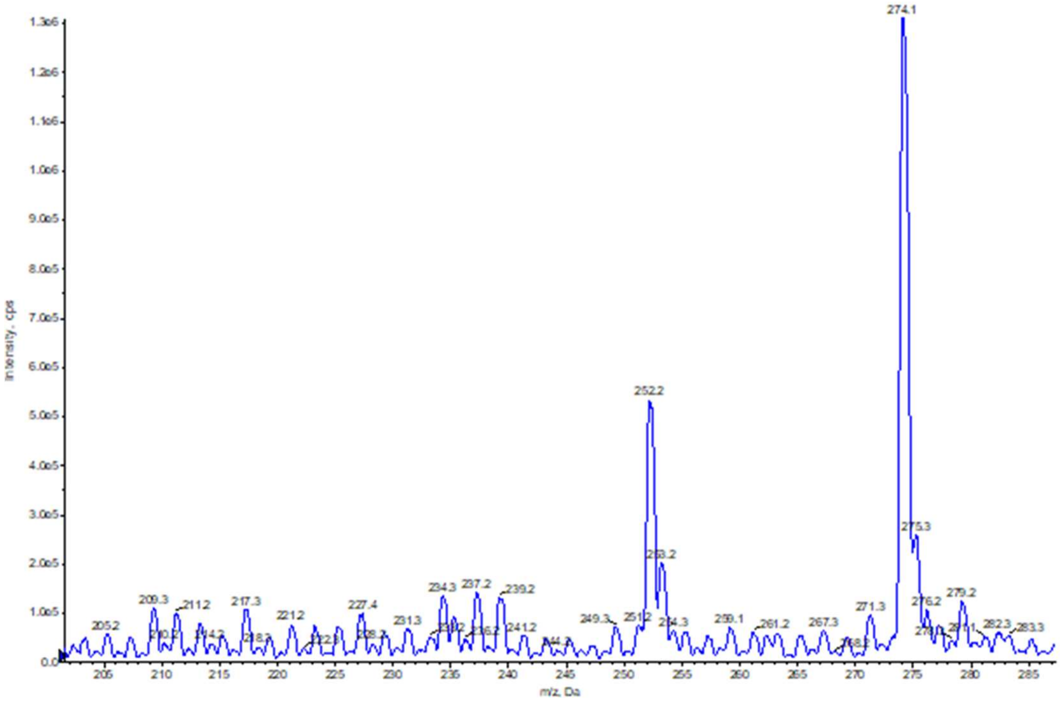

Figure S1. +Q1 mode (without fragmentation)

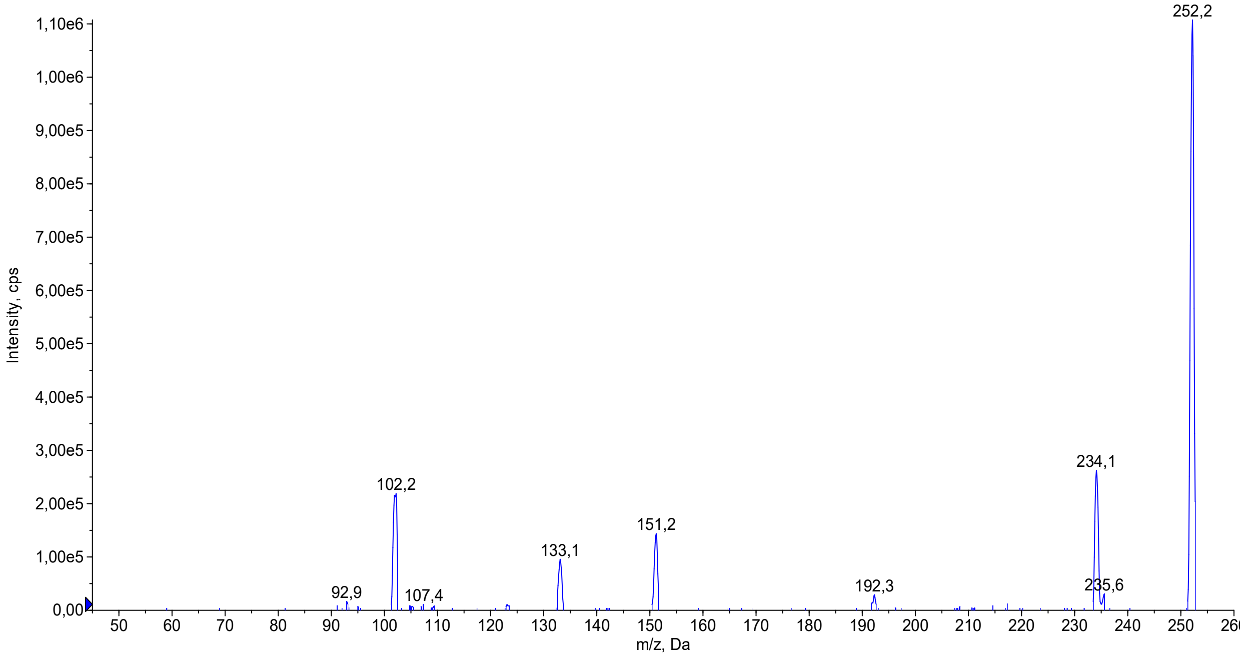

Figure S2. Fragmentation mass spectrum of the protonated form $[M+H]^+$ with m/z=252.1 at low energy (CE = 5 V)

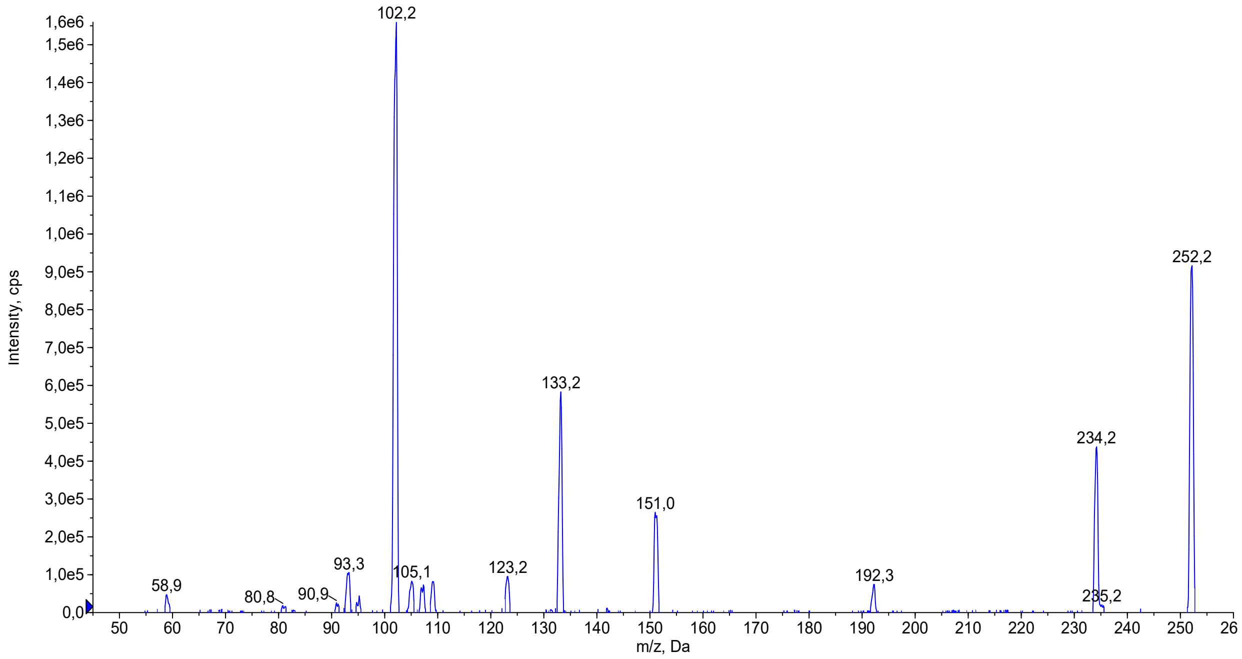

Figure S3. Fragmentation mass spectrum of the protonated form $[M+H]^+$ with m/z=252.1 at medium energy (CE = 15 V).

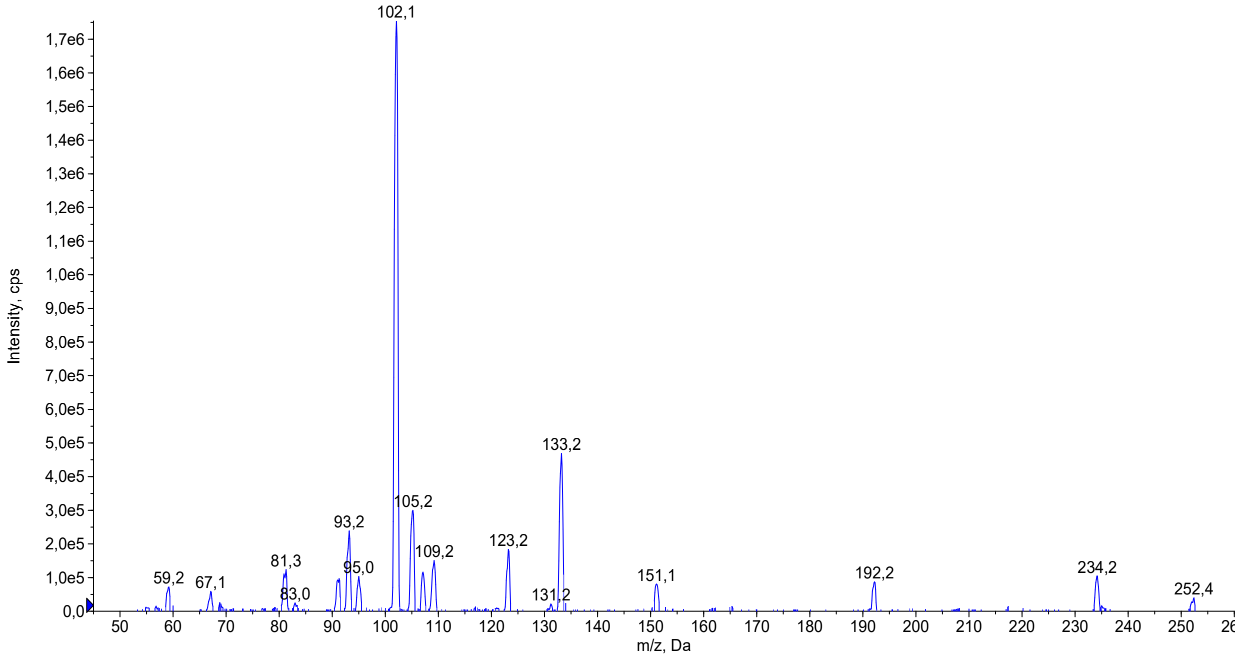

Figure S4. Fragmentation mass spectrum of the protonated form $[M+H]^+$ with m/z=252.1 at high energy (CE = 25 V).

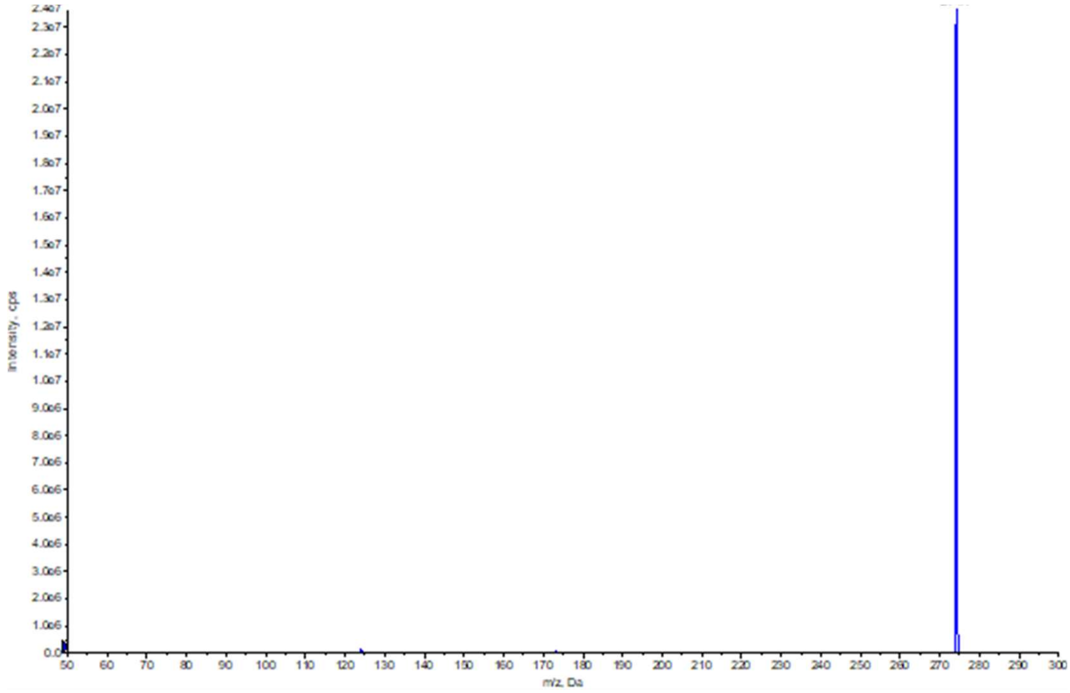

Figure S5. Fragmentation mass spectrum of the adduct $[M+Na]^+$ with m/z=274.1 at low energy (CE = 5 V)

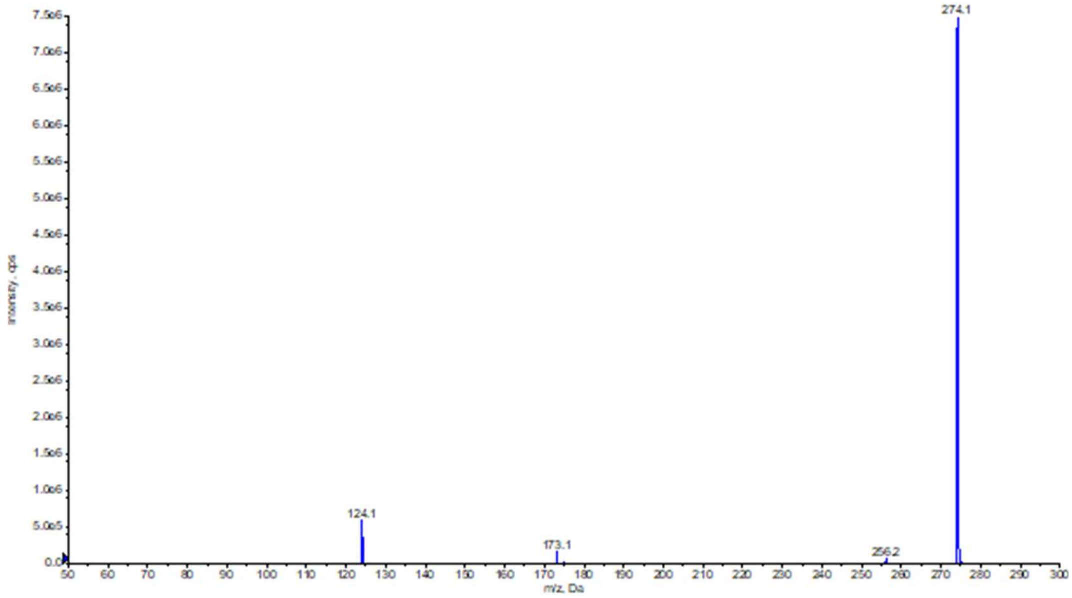

Figure S6. Fragmentation mass spectrum of the adduct $[M+Na]^+$ with m/z=274.1 at medium energy (CE = 15 V)

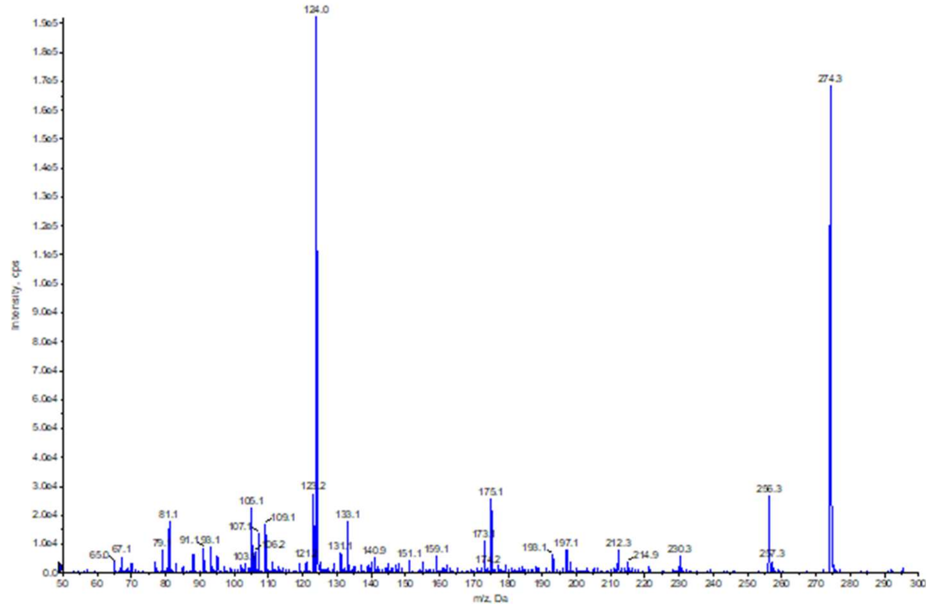

Figure S7. Fragmentation mass spectrum of the adduct $[M+Na]^+$ with m/z=274.1 at medium energy (CE = 25 V)

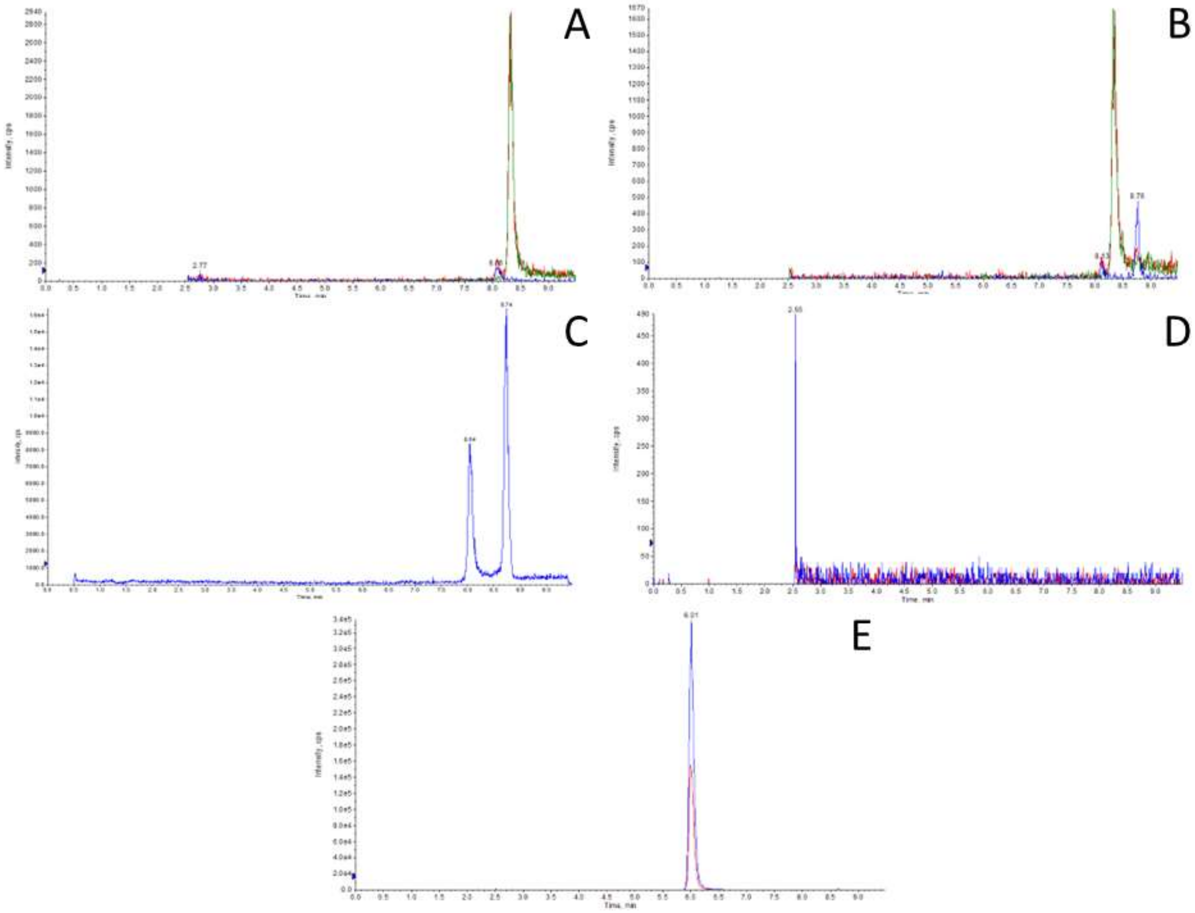

Figure S8. Chromatograms of PA-96 transitions (*m/z* 252.2→102.1/133.1/151.2) of blank blood (A) and LLOQ sample (B). Extracted ion chromatogram of PA-96 of the transition 252.2→102.1 (C). Chromatograms of 2-Ad (*m/z* 152.3→93.1/107.2) in blank blood (D) and the LLOQ sample (E).

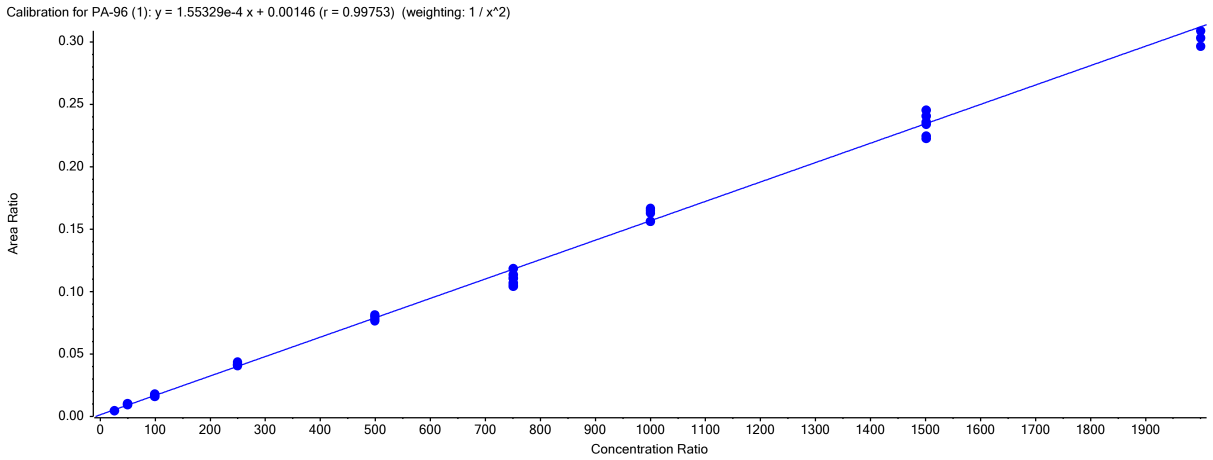

Figure S9 Calibration curve in the range of 25-2000 ng/mL

Table S1. Recovery and matrix effect values

QC sample (ng/mL)	Mean recovery ± SD, %	Matrix factor ± SD
LLOQ (25)	53 ± 4	0.70±0.03
HQC (1500)	69.5 ± 1.2	0.448±0.013

*SD – standard deviation

Table S2. Accuracy and precision for PA-96 quantified in the range of 25-2000 ng/mL.

	LLOQ 25 ng/mL		Low 75 ng/mL		Medium 750 ng/mL		High 1500 ng/mL	
	%nominal ±SD*	%Er**	%nominal ±SD	%Er	%nominal ±SD	%Er	%nominal ±SD	%Er
Day 1	97±3	-3.1	99±4	-0.80	112±3	+12	112±4	+12
Day 2	73±11	-27	101±4	+1.0	94±4	-5.7	88.6±2.1	-11
Day 3	88.7±2.5	-11	88±6	-12	112±3	+12	90.1±2.1	-9.9
Inter-day	86±6	-14	96±5	-4.0	106±3	+6.0	97.0±2.7	-3.0

*SD – standard deviation, %

**Er – percentage relative error

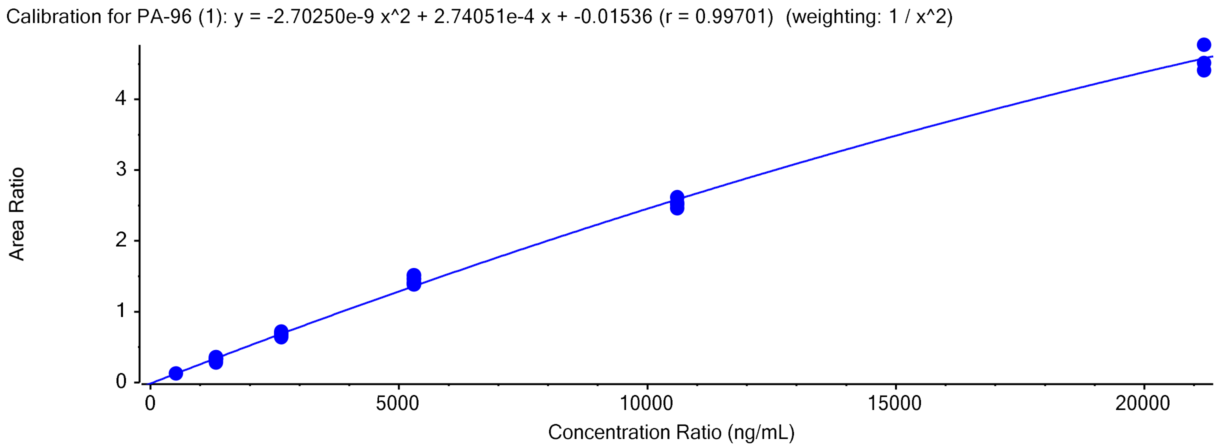

Figure S10. Calibration curve, where x – PA-96 concentration in blood, Y – the ratio of PA-96/IS in 0.9-21200 ng/mL range

Table S3. Accuracy and precision calculated for the high-concentration range calibration curve

Quality control sample and concentration (ng/mL)	Accuracy, %	CV, %
LLOQ (530)	100.5	4.2
QCL (1760)	95.7	4.4
QCM (8800)	96.2	3.9
QCH (17100)	98.5	11.6

Table S4. Pharmacokinetic parameters of PA-96 after p.o and *i.v.* administration to mice at different doses and regimens, *mean* ± *SEM*

PK parameter and dose	Single *p.o.*		Double *p.o.* (2×5 mg/kg)		Single i.v. injection	
	5 mg/kg (n=4)	10 mg/kg (n=4)	1st (n=6)	2nd (n=6)	1 mg/kg (n=6)	10 mg/kg (n=3)
T_{max}, min	6.3 ± 1.3	8.8 ± 1.3	10.0 ± 2.2	248.3 ± 2.5	3.3 ± 0.3	3
C_{max}, ng/mL	36 ± 8	588 ± 71	60 ± 15	58 ± 13	$(3.9 \pm 0.3)\times10^{2}$	$(7 \pm 3)\times10^{3}$
$t_{1/2}$, min	44 ± 13	15.2 ± 1.2	21 ± 4	19 ± 3	37 ± 10	26.1 ± 2.9
AUC_{0-t}, ng/mL*min	$(1.7 \pm 0.3)\times10^{3}$	$(1.8 \pm 0.4)\times10^{4}$	$(1.5 \pm 0.3)\times10^{3}$	$(8.1 \pm 2.3)\times10^{3}$	$(5.1 \pm 0.6)\times10^{3}$	$(1.3 \pm 0.7)\times10^{5}$
$Vz/F_{\_obs}$, (mg)/(ng/mL)	$(1.9 \pm 0.5)\times10^{-1}$	$(13.7 \pm 2.7)\times10^{-3}$	0.108 ± 0.025	0.025 ± 0.004	$(1.1 \pm 0.4)\times10^{-2}$	$(0.40 \pm 0.24)\times10^{-2}$
$Cl/F_{\_obs}$, (mg)/(ng/mL)/min	$(3.3 \pm 0.7)\times10^{-3}$	$(6.4 \pm 1.5)\times10^{-4}$	$(4.0 \pm 0.8)\times10^{-3}$	$(0.79 \pm 0.14)\times10^{-3}$	$(2.01 \pm 0.19)\times10^{-4}$	$(0.10 \pm 0.05)\times10^{-3}$
